# Rs964184 (APOA5-A4-C3-A1) Is Related to Elevated Plasma Triglyceride Levels, but Not to an Increased Risk for Vascular Events in Patients with Clinically Manifest Vascular Disease

**DOI:** 10.1371/journal.pone.0101082

**Published:** 2014-06-30

**Authors:** Anton P. van de Woestijne, Yolanda van der Graaf, Paul I. W. de Bakker, Folkert W. Asselbergs, Wilko Spiering, Frank L. J. Visseren, for the SMART Study Group

**Affiliations:** 1 Department of Vascular Medicine, University Medical Center Utrecht, Utrecht, The Netherlands; 2 Julius Center for Health Sciences and Primary Care, University Medical Center Utrecht, Utrecht, The Netherlands; 3 Department of Medical Genetics, University Medical Center Utrecht, Utrecht, The Netherlands; 4 Broad Institute of Harvard and MIT, Cambridge, Massachusetts, United States of America; 5 Division of Genetics, Department of Medicine, Brigham and Women's Hospital, Harvard Medical School, Boston, Massachusetts, United States of America; 6 Department of Cardiology, Division Heart and Lungs, University Medical Center Utrecht, Utrecht, The Netherlands; 7 Durrer Center for Cardiogenetic Research, ICIN-Netherlands Heart Institute, Utrecht, The Netherlands; University of Milano, Italy

## Abstract

**Background:**

Single nucleotide polymorphisms in the APOA5-A4-C3-A1 gene complex are associated with elevated plasma triglycerides and elevated vascular risk in healthy populations. In patients with clinically manifest vascular disease, hypertriglyceridemia and metabolic syndrome are frequently present, but the contribution of these single nucleotide polymorphisms to plasma triglycerides, effect modification by obesity and risk of recurrent vascular events is unknown in these patients.

**Methods:**

Prospective cohort study of 5547 patients with vascular disease. Rs964184 (APOA5-A4-C3-A1 gene complex) was genotyped, and we evaluated the relation with plasma lipid levels, presence of metabolic syndrome and the risk for new vascular events.

**Results:**

The minor allele of rs964184 was strongly associated with log plasma triglycerides (β 0.12; 95%CI 0.10-0.15, p = 1.1*10^−19^), and was also associated with 0.03 mmol/L lower high-density lipoprotein-cholesterol (95%CI 0.01–0.04), and 0.14 mmol/L higher non-high-density lipoprotein-cholesterol (95%CI 0.09–0.20). The minor allele frequency increased from 10.9% in patients with plasma triglycerides <1 mmol/L to 24.6% in patients with plasma triglycerides between 4 and 10 mmol/L. The relation between rs964184 and plasma triglycerides was modified by body mass index in patients with one minor allele (β 0.02; (95%CI −0.04–0.09) if body mass index <24 kg/m2, β 0.17 (95%CI 0.12–0.22) if body mass index >27 kg/m2, p for interaction = 0.02). The prevalence of the metabolic syndrome increased from 52% for patients with two copies of the major allele to 62% for patients with two copies of the minor allele (p = 0.01). Rs964184 was not related with recurrent vascular events (HR 0.99; 95%CI 0.86–1.13).

**Conclusion:**

The single nucleotide polymorphism rs964184 (APOA5-A4-C3-A1) is associated with elevated plasma triglycerides concentrations in patients with clinically manifest vascular disease. In carriers of one minor allele, the effect on plasma triglycerides was modified by body mass index. There is no relation between rs964184 and recurrent vascular events in these patients.

## Introduction

High plasma triglyceride (TG) levels and low high-density lipoprotein-cholesterol (HDL-c) levels are common among patients with vascular disease and may contribute to residual risk after treatment of other risk factors [1], [2]. Both genetic and environmental factors independently influence the plasma TG level [3] and common genetic variants combined with metabolic factors together lead to elevated TG levels [4]. Genome-wide association studies (GWAS) have identified multiple single nucleotide polymorphisms (SNPs) associated with plasma TG levels [5]–[9]. Of these SNPs, rs964184(APOA5-A4-C3-A1) was also related with the presence of coronary artery disease in a large GWAS for coronary artery disease [10], although very recently also rs2954029(TRIB1) and rs264(LPL) were identified in a GWAS for coronary artery disease [11]. Rs964184 (exact chromosomal position: 11q23.3:116648917) is located near the APOA5-A4-C3-A1 gene complex (MIM 606368, MIM 107690, MIM 107720, MIM 107680), a region with several polymorphisms strongly associated with plasma TG levels [5], [7]–[9]. The nearest gene is ZNF259 (zinc finger protein 259, MIM 603901), located 359 basepairs 3′ from rs964184.

GWAS do not consider effect modification between metabolic factors and SNPs. However, this may be relevant for SNPs associated with plasma TG, since the effect of other SNPs in the APOA5-A4-C3-A1 gene cluster has been shown to depend on secondary factors such as obesity and alcohol [12], [13]. Interactions between genotype and a second factor could be important to determine the individual metabolic consequences of obesity. Overweight and obesity, the metabolic syndrome and the consequent hypertriglyceridemia are common in patients with clinically manifest vascular disease [14]. Therefore, the effect of rs964184 may be different in this population, in which genetically elevated plasma TG are of clinical interest. Furthermore, it is unclear whether a small increase in plasma TG has any effect on the risk for recurrent vascular events in these patients, since all patients with clinically manifest vascular disease are already at high risk for recurrent events and are treated accordingly.

In the present cohort study, we investigated the association between rs964184 and plasma TG levels in patients with vascular disease and whether this association was modified by body mass index. Furthermore, we evaluated the association between rs964184 and clinical parameters such as the metabolic syndrome and being at treatment lipid targets, as well as the risk for occurrence of new vascular events.

## Methods

### Ethics Statement

All patients gave written informed consent, and the Institutional Review Board of the University Medical Center Utrecht approved the study.

### Patients

Data were used from the Second Manifestations of ARTerial disease (SMART) cohort, a prospective, ongoing cohort study, designed to establish the presence of concomitant arterial diseases and risk factors for atherosclerosis in patients with known arterial disease or a cardiovascular risk factor. Patients newly referred to the University Medical Center Utrecht with known arterial disease or cardiovascular risk factors (hyperlipidemia, hypertension or diabetes) are eligible for inclusion. After informed consent, patients underwent a vascular screening protocol including a health questionnaire, laboratory measurements and physical examination. A detailed description of this study has been published previously [15].

For the present study, we used data of 5547 patients, enrolled in the SMART study between September 1996 and March 2011, with either a history or recent diagnosis of clinically manifest arterial disease: coronary artery disease (CAD) (n = 3348), cerebrovascular disease (n = 1597), peripheral artery disease (n = 1115) and/or aneurysm of the abdominal aorta (AAA) (n = 495). Patients could be classified into more than 1 disease category. Patients with plasma TG ≥10 mmol/L (n = 15) were excluded since this most likely results from a rare genetic cause.

### Follow-up

Follow-up duration was defined as the period between study inclusion and first cardiovascular event or death from any cause, date of loss to follow-up or the preselected date of 1 March 2011. During follow-up, all study participants received a questionnaire every 6 months to obtain information about hospitalizations and outpatient clinic visits. If a participant reported a possible event, all available relevant data were collected. Death of a participant was reported by relatives, the general practitioner or the specialist who treated the participant, after which all available relevant data were collected regarding the cause of death. All events were classified independently by three members of the SMART Study Endpoint Committee, comprising physicians from different departments. Outcomes of interest for this study were vascular death, myocardial infarction and a composite of myocardial infarction, ischemic stroke and vascular death.

### Laboratory assessment

Baseline lipid levels were obtained from fasting patients. Plasma total cholesterol and TG were measured using commercial enzymatic dry chemistry kits (Johnson and Johnson). HDL-c in plasma was determined using a commercial enzymatic kit (Boehringer-Mannheim) after precipitation of LDL-c and VLDL-c with sodium phosphotungstate magnesium chloride. LDL-c was calculated using the Friedewald formula.

### Genotyping

Wet-lab genotyping genotyping for SNP analysis was carried out by KBiosciences, Hertfordshire, UK (www.kbioscience.co.uk), whose personnel were blinded to patient status, using their proprietary KASPar PCR technique and Taqman Genotype calling was carried out using an automated system, the results of which were checked manually by study personnel using SNPviewer software. Individuals with a low overall genotyping rate were removed from the study (n = 73). Rs964184 was used for the present study, for 112 patients (2.0%), rs964184 genotype was missing, resulting in 5547 patients for the current analyses.

### Data analyses

Linear regression models were used to evaluate the association between rs964184 and several lipid parameters (plasma triglycerides, HDL-c, nonHDL-c, apoB), with adjustment for age and sex. Since mainly nonHDL-c and apoB are affected by lipid-lowering therapy, these analyses were also adjusted for lipid-lowering medication. ApoB was only available for patients included from 2006 onwards (2133 patients). Furthermore, to investigate whether the association between rs964184 and plasma TG is modified by metabolic consequences of obesity, we included an interaction term with body mass index in a separate model.

To determine whether rs964184 is associated with metabolic syndrome prevalence, we used a logistic regression model, adjusted for age and sex. Additionally, in patients using lipid-lowering medication, a logistic regression model adjusted for age and sex was used to evaluate whether rs964184 is associated with the proportion of patients who were on treatment target for LDL-c (2.5 mmol/L), nonHDL-c (3.3 mmol/L) or apoB (100 mg/dL) [16].

A Cox proportional hazards model was used to detect an effect of rs964184 on vascular events in patients with known vascular disease, after testing the proportional hazards assumption. This model was adjusted for age and sex. Furthermore, in a second model additional adjustment for plasma TG was performed, to identify whether a possible association between rs964184 and vascular events could be explained by plasma TG level.

For the analyses, the open source software package R 2.13 was used. A p-value of <0.05 was considered statistically significant for all analyses.

## Results

### Baseline characteristics

Baseline characteristics according to rs964184(APOA5-A4-C3-A1) genotype are shown in Table 1. Plasma TG levels are higher and HDL-c levels are lower for the heterozygotes and the homozygotes for the G allele. Furthermore, coronary artery disease is slightly more present in carriers of the G allele, whereas cerebrovascular disease is slightly less present. The overall minor allele frequency was 14.2%.

**Table 1:** Baseline characteristics stratified per rs964184 genotype.

	CC	CG	GG
	(n = 4100)	(n = 1313)	(n = 134)
Age (years)	60.0 ± 10.4	60.1 ± 10.3	61.0 ± 9.7
Male sex, n (%)	3048 (74)	982 (75)	101 (75)
BMI (kg/m^2^)	26.8 ± 3.9	26.7 ± 4.0	27.1 ± 4.0
Total cholesterol (mmol/L)	4.9 ± 1.2	5.0 ± 1.3	5.2 ± 1.3
Triglycerides (mmol/L)	1.6 ± 1.0	1.9 ± 1.2	2.2 ± 1.3
LDL-c (mmol/L)	3.0 ± 1.0	2.9 ± 1.1	3.0 ± 1.1
HDL-c (mmol/L)	1.23 ± 0.37	1.20 ± 0.35	1.19 ± 0.31
Systolic blood pressure (mmHg)	141 ± 21	141 ± 21	141 ± 21
Diastolic blood pressure (mmHg)	82 ± 11	82 ± 11	81 ± 11
Metabolic Syndrome, n (%)	2139 (52)	718 (55)	83 (62)
Type 2 DM, n (%)	686 (17)	211 (16)	29 (22)
eGFR* (mL/min/1.73 m^2^)	76 ± 18	76 ± 18	73 ± 19
Localisation of vascular disease, n (%):			
- coronary artery disease	2449 (60)	813 (62)	86 (64)
- cerebrovascular disease	1198 (29)	365 (28)	34 (26)
- peripheral artery disease	824 (20	260 (20)	31 (23)
- aneurysm of abdominal aorta	367 (9)	119 (9)	9 (7)
Lipid-lowering medication, n (%)	2596 (63)	869 (66)	90 (67)
Antihypertensive medication, n (%)	3003 (73)	974 (74)	105 (78)
Current smoking, n (%)	1383 (34)	388 (30)	43 (32)
Current alcohol use, n (%)	1993 (49)	661 (50)	61 (46)

* Glomerular Filtration Rate, estimated by the Modification of Diet in Renal Disease (MDRD) equation.

**Figure 1:**
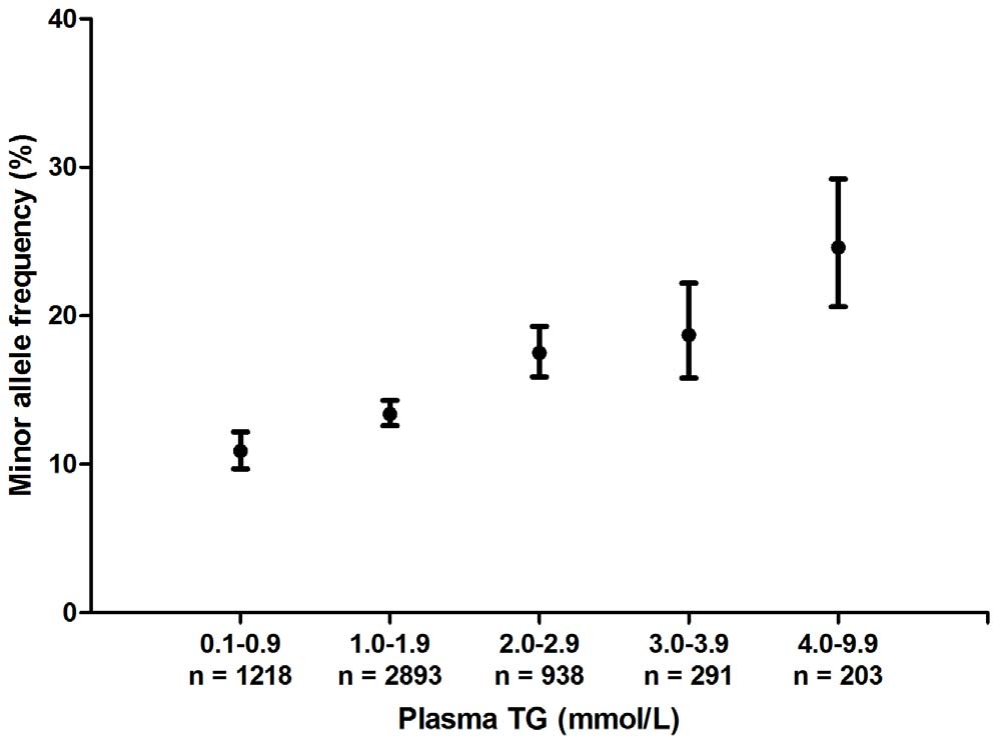
Relation between minor allele frequencies and levels of plasma TG. Rs964184 minor allele frequency (95% confidence interval) at increasing levels of TG. N indicates the number of patients with a plasma TG level in this category.

In figure 1 it is shown that the minor allele frequency increases with increasing levels of plasma TG, being 10.9% in patients with plasma TG levels between 0 and 1 mmol/L and 24.6% in patients with plasma TG levels between 4 and 10 mmol/L.

### Relation between rs964184 and plasma lipids

Rs964184 was strongly associated with plasma TG levels, with log(TG) being 0.12 (95%CI 0.10-0.15, p = 1.1*10^−19^) higher per minor allele (Table 2). The association with HDL-cholesterol was less strong, HDL-c levels were 0.03 (95%CI −0.04– −0.01) mmol/L lower per minor allele. The SNP was also associated with increased levels of nonHDL-c (0.14 mmol/L; 95%CI 0.09 – 0.20) and apoB (0.03 g/L, 95% CI 0.01 – 0.05).

**Table 2:** Relation between rs964184 (per minor allele) and lipid levels.

	Beta (95% CI)	P
Log(TG)	0.12 (0.10 – 0.15)	1.1*10^−19^
HDL-c	−0.03 (−0.04 – −0.01)	0.007
NonHDL-c*:	0.14 (0.09 – 0.20)	6.3*10^−7^
LDL-c	0.02 (−0.03 – 0.07)	0.34
ApoB*	0.03 (0.01 – 0.05)	0.004

Linear regression adjusted for age and sex.

*Additionally adjusted for lipid-lowering medication.

**Figure 2:**
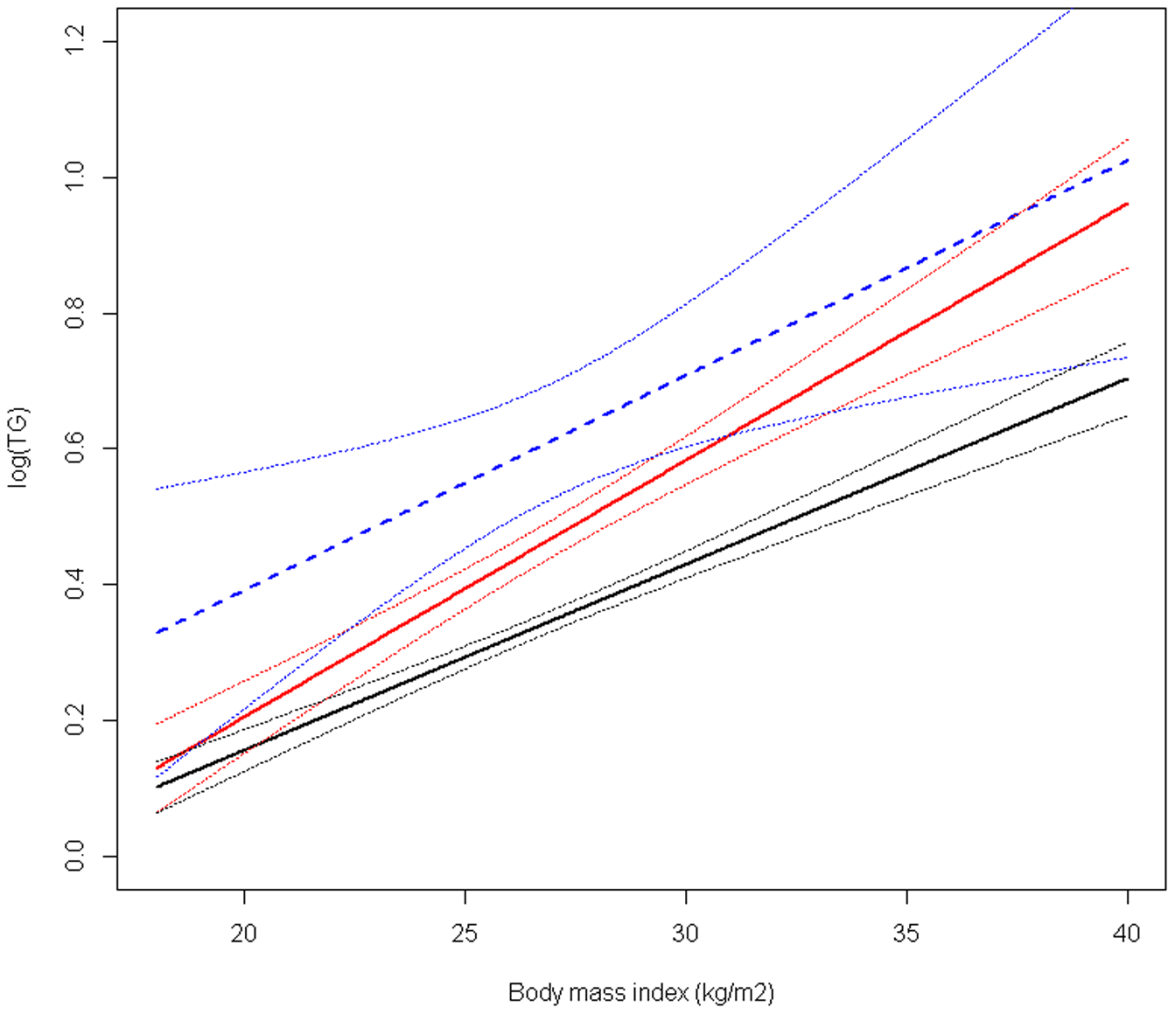
Association between body mass index and plasma log(TG), stratified by rs964184 genotype. Black line: CC genotype, red line: CG genotype, blue line: GG genotype. Dotted lines indicate 95% confidence interval.

The relation between rs964184 and plasma TG was modified by BMI (p for interaction = 0.03, Figure 2). When stratified for genotype, only the effect of the CG genotype was modified by BMI (p for interaction = 0.02). Including a quadratic term for BMI did not change these results. In the patients heterozygous for the minor allele with a BMI<24 kg/m^2^, log(TG) was increased with 0.02 (95%CI −0.04 − 0.09) compared with patients without the minor allele, whereas the log(TG) was 0.13 (95%CI 0.08–0.18) higher in patients with a BMI≥24 kg/m2 and <27 kg/m2 and 0.17 higher (95%CI 0.12–0.22) in patients with a BMI ≥ 27 kg/m2. Conversely, per 1 kg/m^2^ increase in BMI, log(TG) increased with 0.038 (95%CI 0.031–0.045) in heterozygous patients compared with 0.028 (95%CI 0.024–0.032) and 0.030 (95%CI 0.006–0.054) in patients homozygous for the major or the minor allele, respectively.

### Relation between rs964184 and metabolic syndrome and lipid treatment targets

The prevalence of the metabolic syndrome is higher in patients with the minor allele. The prevalence was 52% in patients homozygous for the major allele and 62% in patients homozygous for the minor allele, (Odds Ratio of 1.14 (95%CI 1.03–1.27)) (Table 3). Furthermore, the elevated plasma TG levels also results into a decreased proportion of patients who were at the nonHDL-c or apoB treatment target (Table 3), whereas it did not influence the proportion of patients reaching the LDL-c target of <2.5 mmol/L. In patients with two copies of the major allele and using lipid-lowering medication 55% were at the nonHDL-c target of <3.3 mmol/L, whereas in patients with 2 copies of the minor allele and using lipid-lowering medication, this was 47%. For the apoB target, 80% of the patients with two copies of the major allele were at treatment target as opposed to 67% of the patients with two copies of the minor allele.

**Table 3:** Relation between rs964184 genotype and presence of metabolic syndrome or being not at lipid treatment target.

	N (n*)	OR (95% CI)	P
Metabolic syndrome	5547 (2940)	1.14 (1.03 – 1.27)	0.01
Not at LDL-cholesterol target**	3432 (1696)	0.95 (0.83 – 1.08)	0.43
Not at nonHDL-cholesterol target**	3553 (1612)	1.12 (0.98 – 1.27)	0.10
Not at apoB target**	1730 (357)	1.26 (1.01 – 1.57)	0.04

Logistic regression model, adjusted for age and sex.

*Number of patients with metabolic syndrome or LDL-cholesterol/nonHDL-cholesterol/apoB not at target level.

**Only in patients using lipid lowering medication.

### Relation between rs964184 and risk for new vascular events

During 32,200 patient-years of follow-up, a total of 825 vascular events occurred. There was no relation between rs964184 and occurrence of new vascular events (HR 0.99; 95%CI 0.86–1.13; Table 4), vascular mortality (HR 1.09; 95%CI 0.92–1.30) or myocardial infarction (HR 1.00; 95%CI 0.83–1.21). After adjustment for plasma TG, the HRs decreased, consistent with an association between both rs964184 and plasma TG, and between plasma TG and vascular events.

**Table 4:** Relation between rs964184 and risk of new vascular events.

	Myocardial infarction	Vascular mortality	All vascular events
	HR (95%CI)	HR (95%CI)	HR (95%CI)
N (events)	5547 (434)	5547 (479)	5547 (825)
Model 1	1.00 (0.83–1.21)	1.09 (0.92–1.30)	0.99 (0.86–1.13)
Model 2	0.96 (0.79–1.16)	1.05 (0.88–1.25)	0.95 (0.83–1.09)

Hazard ratio (95% confidence interval) estimated with Cox proportional hazard models.

Model 1: adjusted for age and sex.

Model 2: Model 1 + triglyceride plasma level.

## Discussion

The SNP rs964184 in the APOA5-A4-C3-A1 region is associated with plasma TG level in patients with clinically manifest vascular disease. In patients with one minor allele, this association is modified by BMI. Furthermore, rs964184 was related to the presence of metabolic syndrome, but was not related to the risk for new vascular events.

Previous studies have shown that several polymorphisms in or near the APOA5-A4-C3-A1 gene complex, among which rs964184, are associated with plasma TG level [5], [7], [8] and vascular risk [10] in healthy populations. To our knowledge, no studies have specifically addressed this issue in patients with vascular disease. Among patients with clinically manifest vascular disease, the metabolic syndrome and elevated TG levels are common [14], and the results of the present study show that rs964184 is associated with plasma TG in these patients, as well as with HDL-c, nonHDL-c and apoB. Although in this population of patients with manifest vascular disease the average TG levels may be higher than in the general population, the magnitude of the association is still similar to the association reported in the general population [5], [7], [8]. Due to the higher level of TG-rich particles, the LDL-c levels are less accurate in estimating the atherosclerotic lipoprotein particle burden, since there is an higher concentration of atherogenic TG-rich remnant particles and since higher TG plasma levels lead to development of atherogenic small dense LDL particles [17]. Therefore, the nonHDL-c levels or apoB levels may better reflect the cardiovascular risk since they include all atherogenic particles. This is demonstrated in the present study, indicating a smaller chance to be at the apoB treatment target associated with the minor allele, despite a similar proportion of patients being at the LDL-c treatment target.

The prevalence of the metabolic syndrome in carriers of the minor allele was higher and this finding is concordant with results in the general population, although the relation is less strong than in the general population [18]. This may be due to the already higher prevalence of the metabolic syndrome among patients with manifest vascular disease, attenuating the contrast between groups with a different genotype. Nevertheless, the association of rs964184 with the metabolic syndrome persists, showing that also in this high risk population rs964184 does contribute to the prevalence of the metabolic syndrome. Since this SNP only selectively influences TG and HDL-c level, whereas the metabolic syndrome is seen as a clustering of risk factors related to obesity and insulin resistance, the contribution of rs964184 to the prevalence of the metabolic syndrome may be regarded as obscuring the real common basis of metabolic syndrome [18]. However, since the difference in plasma TG between patients homozygous for the major allele and patients with one minor allele depends on BMI, this will still lead to clustering of obesity and plasma TG, in which obesity is the trigger for expression of increased TG level. Furthermore, these type of interactions may also explain why some obese patients are less vulnerable to develop the metabolic consequences of obesity, as they may have a favorable genetic background. This could be one of the underlying explanations for the existence of metabolically healthy obese patients [19]. Conversely, the TG levels for carriers of one minor allele may be similar to the homozygote wild type patients, provided that their BMI is low. This is in line with the model that common genetic variants combined with modifier variables (genes or other factors such as obesity and insulin resistance) together lead to elevations in plasma TG levels [4] and it is in accordance with the mechanism by which also mutations in the near APOA5 gene cause hypertriglyceridemia, requiring a second factor to express the clinical phenotype [20]. Although genotype cannot be changed, the phenotype may be influenced by reducing bodyweight, and our results underscore the importance of advocating weight loss for overweight patients with hypertriglyceridemia.

Rs964184 was not associated with recurrent vascular events in the present study. A reason for the apparent absence of the association between rs964184 and vascular events could be the sample size of the study, since especially patients carrying two copies of the minor allele were infrequent and hence also few events occurred in this group (n = 134 with 20 vascular events during follow up). The relatively small increase in risk of vascular events of 13% associated with the presence of rs964184 in the general population, as found in the Cardiogram GWAS, may be too small to be relevant in high-risk patients. Patients with clinically manifest vascular disease not having the minor allele may have a higher burden of other risk factors leading to a first vascular event [21]. This can also be observed from the baseline table, as the frequency of the minor allele is increased in patients with coronary artery disease compared to patients with cerebrovascular disease. This may indicate that rs964184 (and hence plasma TG) is a more important risk factor for coronary artery disease than for cerebrovascular disease, and that patients without the minor allele have other risk factors, predisposing more to cerebrovascular disease.

The present results do not justify genotyping TG-associated SNPs in clinical practice, since they do not add clinically significant information to readily available characteristics and the known risk profile. However, once large scale genotyping is available at low costs, these results show that individualized treatment or lifestyle advices could be given since the effects of influencing secondary factors may depend on the genetic background.

Strengths of the study are the observational cohort reflecting clinical reality, and the extensive phenotyping of all patients according to standardized procedures, ascertainment of vascular events in this cohort and low proportion (<4%) lost to follow up. A limitation of this study is the genotyping of only one TG-associated SNP. Furthermore, the use of lipid-lowering medication during follow up may have changed and may thus potentially influence the associations between the SNP and plasma TG or vascular events. However, the effect of statins on plasma TG is small and adjustment for baseline use of lipid-lowering medication did not influence the results. Drugs specifically targeting plasma TG, such as fibrates, were used by very few patients (0.8%). In addition, the group of patients homozygote for the minor allele was relatively small (134 patients), which could in theory result in a lack of power to detect the effect modification by BMI in this group of patients, hence the absence of effect modification in this group may be a false negative finding. However, as was explained above, the absence of effect modification by BMI in this group may also be plausible from a theoretic point of view. Finally, the results of the present study were not replicated in an independent cohort, which would strengthen our conclusions. However, since this study investigates one SNP known to be associated with plasma TG and vascular events in the general population, it is less prone to finding spurious associations than studies in which a large number of SNPs is investigated for new associations with disease traits.

In conclusion, rs964184 in the APOA5 region is associated with elevated TG plasma levels and presence of the metabolic syndrome in patients with clinically manifest vascular disease. Although this association translated into a decreased probability to be at apoB treatment target and a trend towards being not at nonHDL-c treatment target, there was no relation between rs964184 and the risk for new vascular events in these patients.
